# Prevalence of *Helicobacter pylori* and Its Associated Factors among Healthy Asymptomatic Residents in the United Arab Emirates

**DOI:** 10.3390/pathogens8020044

**Published:** 2019-04-01

**Authors:** Ghalia Khoder, Jibran Sualeh Muhammad, Ibrahim Mahmoud, Sameh S. M. Soliman, Christophe Burucoa

**Affiliations:** 1 Department of Pharmaceutics and Pharmaceuticals Technology, College of Pharmacy, University of Sharjah, Sharjah 27272, UAE; 2 Sharjah Institute for Medical Research, University of Sharjah, Sharjah 27272, UAE; 3 Department of Basic Medical Sciences, College of Medicine, University of Sharjah, Sharjah 27272, UAE; 4 Department of Family Medicine and Behavioral Sciences, College of Medicine, University of Sharjah, Sharjah 27272, UAE; iabdelmahmoud@sharjah.ac.ae; 5 Department of Medicinal Chemistry, College of Pharmacy, University of Sharjah, Sharjah 27272, UAE; ssoliman@sharjah.ac.ae; 6 Laboratoire de bactériologie, Hygiène, EA 4331 LITEC, CHU de Poitiers, Université de Poitiers, Poitiers 86000, France; Christophe.BURUCOA@chu-poitiers.fr

**Keywords:** prevalence, *H. pylori*, stool, Premier Platinum HpSAT, United Arab Emirates

## Abstract

The United Arab Emirates (UAE) has been under continuous populational influences from Asia, Europe, and Africa, making it an ideal site for epidemiological studies on *Helicobacter pylori*. However, there has been a paucity of well-designed prevalence studies on *H. pylori* from UAE. The aim of this study was to determine the prevalence of *H. pylori* and its associated risk factors in the UAE. A prospective cross-sectional study was conducted on healthy asymptomatic residents of UAE. Socio-demographic, lifestyle, and gastrointestinal characteristics of participants were obtained through a questionnaire in parallel within the stool sample collection. A total of 350 participants were included in this study and were tested for *H. pylori* using the stool antigen test (Premier Platinum HpSAT). Out of the total tested study participants, 41% were found to be *H. pylori*-infected. Logistic regression analysis has shown a significant association between *H. pylori* infection and gender, age, ethnicity, profession, domestic overcrowding, source of drinking water, and gastrointestinal characteristics of participants. Based on the results from this study, we suggest that preventive measures against *H. pylori* infection should be considered worthy by public health authorities.

## 1. Introduction

Helicobacter pylori (*H. pylori*) is a gram-negative microaerophilic bacterium that colonizes the gastric mucosa of more than half of the world’s population with high geographic variability [1]. *H. pylori* infection is generally acquired during childhood and persists life-long in the absence of treatment with antibiotics. Most of the infected individuals remain asymptomatic for a long period. As a result, long-term colonization of *H. pylori* can damage the gastric mucosa causing various diseases of the upper gastrointestinal tract such as chronic gastritis, peptic ulcer, and gastric malignancies, particularly gastric cancer and gastric mucosa-associated lymphoid tissue (MALT) lymphoma [2,3]. *H. pylori* has been recognized as a Class I carcinogen by the International Agency for Research on Cancer [4] and as one of the strongest known risk factors for gastric malignancies [5,6,7,8]. Approximately 89% of all gastric cancers are attributed to *H. pylori* infection and the eradication of this infection has known to reduce gastric cancer incidence [9,10]. Gastric cancer is ranked sixth in incidence and second in mortality among all cancers worldwide based on the most recent global cancer statistics in 2018 [11]. In UAE, the Health Authority of Abu Dhabi (HAAD) ranked gastric cancer as seventh among the top 10 cancers in 2011. Even though gastric cancer incidence and mortality decreased substantially over the last two decades in most countries worldwide, there is still a need to control *H. pylori* infection to further reduce the burden of gastric cancer [12,13]. It is now well established that *H. pylori* screening and eradication is relatively cost-effective in reducing the burden of gastric cancer and peptic ulcer in high prevalence populations [14]. Implementation of eradication strategies requires up-to-date information regarding the prevalence of *H. pylori* and its associated factors. Furthermore, *H. pylori* infection has been associated with several extra-digestive diseases such as iron-deficiency anemia and idiopathic thrombocytopenic purpura [15]. The probable routes of transmission of *H. pylori* are feco-oral, oro-oral, and intra-familial [16], thus rendering the risk factors for *H. pylori* infection closely associated with food and personal hygiene. Other known risk factors associated to *H. pylori* infection include age, socioeconomic status, number of siblings, household crowding, ethnicity, migration from high prevalence regions, infection status of family members, and sanitary facilities [17,18,19]. Diagnosis of *H. pylori* can be performed through invasive (rapid urease test, histology, culture, and PCR) and non-invasive methods (serological tests, stool antigen test, and urea breath test) [20,21,22,23]. The major disadvantage of the invasive tests is that they require endoscopic examination for obtaining the diagnostic sample and hence are difficult to use in epidemiological studies. The *H. pylori* stool antigen test (HpSAT) has improved enormously in the past few years and has gained great interest in *H. pylori* detection [24,25]. Several studies have demonstrated that stool monoclonal tests are reliable for diagnosing *H. pylori* infection and therefore facilitate robust clinical and epidemiological studies, especially in children and toddlers [26,27,28,29].

*H. pylori* prevalence ranges between 85% and 95% in developing countries and between 30 and 50% in developed countries [30,31,32]. The epidemiology of *H. pylori* infection has changed with improvements in sanitation and methods of eradication. However, the prevalence of *H. pylori* is still abundant. In developing countries, the prevalence remains the highest, and this is related to socioeconomic status and levels of hygiene. After the year 2000, the prevalence of *H. pylori* became lower than before in European countries. However, in Asia, the prevalence remains the same [1]. To this date, 4.4 billion individuals globally are estimated to be *H. pylori*-infected [1]. The area with the highest reported prevalence was reported in Africa (70.1%) while the lowest prevalence was reported in Switzerland (18.9%). In Southern Asia, Pakistan and India have shown the highest *H. pylori* prevalence (81% and 63.5%, respectively). In Western Asia, Turkey presented the highest prevalence (77.2%). Among the 62 countries investigated in the most recent global prevalence study in 2018, there were no informative prevalent data about *H. pylori* in UAE. However, in the neighboring countries such as Oman and Saudi Arabia, the prevalence of *H. pylori* was estimated to be 49.1% and 65.9%, respectively [1]. Estimation of *H. pylori* infection very much needs to be explored in UAE. In the past 26 years, only 33 studies related to *H. pylori* infection were performed in UAE (based on the PubMed search). However, only two of those studies investigated the prevalence of *H. pylori* in asymptomatic subjects. Of those, the first study was conducted on a restricted population of low socioeconomic workers [33]. While the second study was conducted on asymptomatic farmers and were compared with non-farmers [34]. The novelty of our study resides in being the first study to estimate the prevalence of *H. pylori* and its associated factors taking into consideration the multinational population existing in the UAE. Understanding the prevalence pattern of *H. pylori* in UAE and its associated factors will aid in prioritizing and customizing public health efforts to better manage the burden of *H. pylori* infections and its associated diseases.

## 2. Results

### 2.1. Socio-Demographic and Lifestyle Characteristics

A total of 350 participants responded to the questionnaires (see supplementary “Questionnaire”), among participants, there were 131 (37%) females and 219 (63%) males. Most of the participants were reported from Sharjah (48%), then Abu Dhabi (33.7%), Ajman (15.4%) and Dubai (3%). Table 1 shows *H. pylori* prevalence for each of these Emirates regions according to gender, ethnicity, and age group. Participants age ranged from 1 to 68 years in age, mostly belonging from the age group of 30 years old and lower (60.6%). Among the age groups, participants aged between 30 years and above constituted the largest group (138, 39.4%), compared to the age group from 2 to 5 years (89, 25.4%). The age group from 0 to 12 months constituted the fewest number of participants (13, 3.7%) (Table 2). 

The ethnicities of the participants were distributed as follows: Arab (163, 46.6%), Asian (165, 47.1%), and African (22, 6.3%). In term of household income, most of the participants had less than AED 10000 (2700 USD) monthly family income (133, 45.4%). The occupation of participants was distributed as following: Employed as a professional (37, 10.6%), employed as an industrial worker (97, 27.7%), employed as a nanny (56, 16%), and student (145, 41.4%). Only 4.4% of the participants were unemployed. All children participants were considered as students since stool samples from this age group were collected from schools and nurseries. More than half of the participants lived with 4 to 6 persons per house (207, 59.1%) compared to participants living with more than 7 persons per house (112, 32%). In term of a number of persons per room, more than half of the participants reported living in close proximity with others, having three persons or above per room (194, 55.4%). In terms of having contact with domestic animals and type of drinking water, most of the participants confirmed no contact with domestic animals (305, 87.1%) and that mineral bottled water was the source of their drinking water (283, 80.9%) (Table 2).

### 2.2. Gastrointestinal Characteristics

When participants were asked about having experienced gastrointestinal disturbances, the majority have reported no previous or current gastrointestinal disturbances (288, 82.3%). Among the few participants having experienced gastrointestinal disturbances, abdominal pain was the major symptom (29, 8.3%), followed by bloating (20, 5.7%). In terms of parent gastric diseases history, the majority did not report any history case of familial gastric diseases (293, 83.6%). Among the few participants having a familial history of gastric diseases, peptic ulcer was reported in the first place (31, 8.9%) compared to other gastric diseases including gastric cancer and MALT lymphoma (Table 2). None of the participants have reported any past, recent, or current intake of Proton Pump Inhibitors (PPI) for *H. pylori* eradication treatment (data not shown in the table).

### 2.3. Prevalence of H. pylori Infection in Asymptomatic Healthy UAE Residents

Analysis of the collected stool samples of the 350 participants has shown that 145 participants were *H. pylori*-infected and therefore the prevalence of *H. pylori* infection was considered 41% including both children and adults. Bivariate analysis has shown that *H. pylori* infection exhibited significant differences across several sociodemographic, lifestyle, and gastrointestinal characteristics (Table 2). Participants from Ajman showed the highest prevalence (35, 64.8%) to *H. pylori* compared to Abu Dhabi, Sharjah, and Dubai. Female participants were more prone to *H. pylori* infection (69, 53%) compared to male participants (76, 35%) with a statistically significant difference (*p* = 0.001). In term of age groups, a statistically significant difference was found between *H. pylori* infection and the age group of participants. The prevalence of *H. pylori* was found to increase significantly with the age of participants (*p* = 0.032). Adults participants aged between 16 and 30 years old showed the highest prevalence for *H. pylori* (37, 52.9%), followed by participants 30 years old and older (63, 46%). 

**Figure 1:**
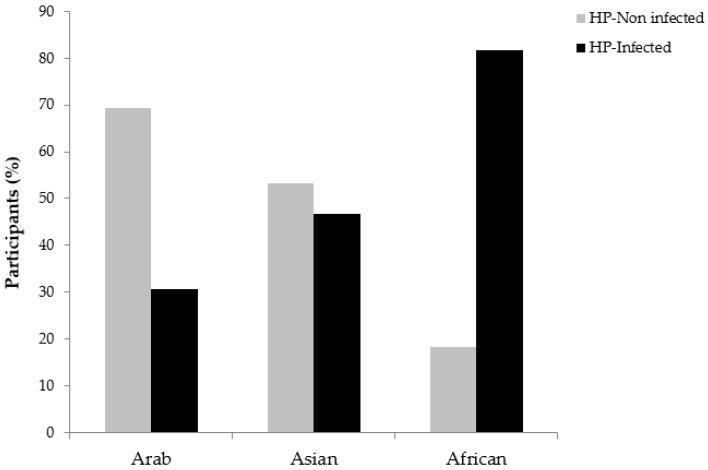
Prevalence of *H. pylori* according to the ethnicity of the 350 healthy asymptomatic residents in the UAE.

Since people of several ethnicities reside in UAE, it was of great interest to investigate which ethnicity is more prone to *H. pylori* infection. A significant statistical difference was found between *H. pylori* infection and the ethnicity of the participants (*p* < 0.0001) (Table 2). African residents in UAE presented the highest prevalence to *H. pylori* (18, 81.8%) compared to Asian (77, 46.7%) and Arab participants (50, 30.7%) (Figure 1). Among the Asian group, participants from India were found more infected by *H. pylori* (39%) followed by participants from Pakistan (23%) and the Philippines (21%). Among African participants, the prevalence of Ethiopian participants presented the highest positivity to *H. pylori* (71%). In Arab participants, Emiratis were the most prone to *H. pylori* infection (55%) compared to other Arab nationalities. All other Arab participants presented a similar prevalence to *H. pylori*. Taking together all *H. pylori*-infected participants, the prevalence was the highest among the Ethiopian nationality (71%) compared to the other nationalities (data not shown). 

**Figure 2:**
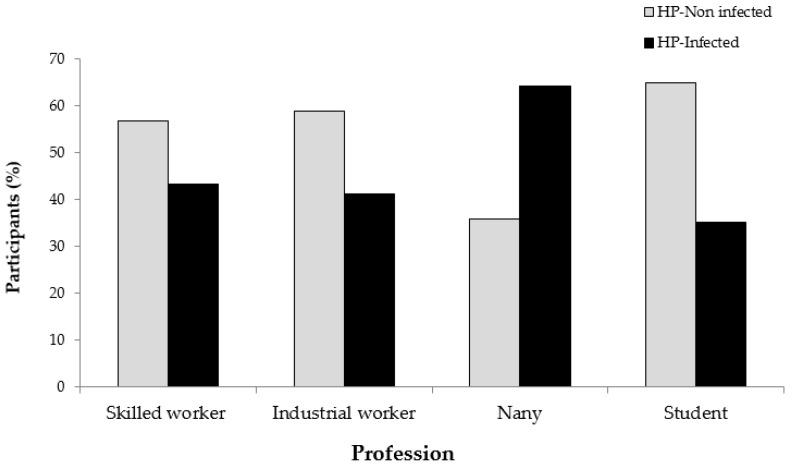
Prevalence of *H. pylori* according to the profession of the 350 healthy asymptomatic residents in the UAE.

In term of participant occupation, a statistically significant difference was found between *H. pylori* and occupation of participants (*p* = 0.001). Among the considered occupations, nannies showed the highest prevalence to *H. pylori* (36, 64.3%) followed by professional workers (16, 43.2%), industrial workers (40, 41.2%), and students (51, 35.2%) (Figure 2).

In term of the number of persons per house, no statistically significant difference was found between *H. pylori* infection and these factors (data not shown). However, the number of persons per room was found to play an important role in *H. pylori* infection with a statistically significant difference (*p* = 0.0001). Half of the participants living in crowded conditions (≥3 members per room) were infected by *H. pylori* (97, 50%) (Table 2).

**Figure 3:**
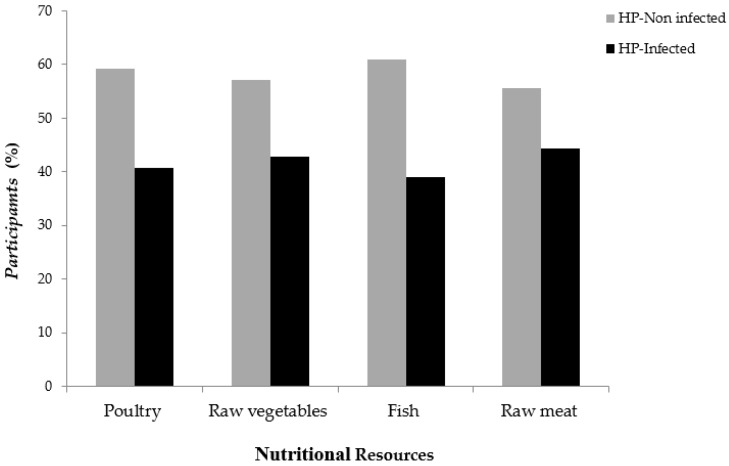
Prevalence of *H. pylori* according to the nutritional resource of the 350 healthy asymptomatic residents in the UAE.

In term of the drinking water source, even though most of the participants confirmed drinking mineral bottled water (283, 80.9%), a statistically significant difference was found between *H. pylori* and tap water as a source of drinking water (*p* = 0.001). More than half of participants drinking tap water were found positive to *H. pylori* (34, 58.6%). In term of gastrointestinal characteristics, even though 82.3% of the involved participants were healthy and asymptomatic without any gastrointestinal disturbances, few participants have mentioned suffering from gastrointestinal disturbances. Interestingly, a statistically significant difference was found between the *H. pylori* infection and the gastrointestinal disturbances (*p* = 0.001). Participants suffering from abdominal pain were found highly prevalent to *H. pylori* (22, 75.9%) followed by bloating (8, 40%). When family history of gastrointestinal diseases was investigated, it was found that participants having one of their parents with previous or current peptic ulcer diseases were highly prevalent to *H. pylori* (28, 90.3%) with a statistically significant difference (*p* < 0.0001). There was no statistically significant difference found between nutritional resources and *H. pylori* infection (Figure 3).

### 2.4. Association Between H. pylori Infection and Socio-Demographic, Lifestyle and Gastrointestinal Characteristics

Binary logistic regression analysis revealed that female participants had a significantly higher likelihood of being diagnosed as *H. pylori-*infected compared to a male participants, Odds ratio (OR) 2.52, 95% CI, 1.16–5.50, *p* = 0.020. Furthermore, Asian and Africans were more likely to have *H. pylori* infection, OR 7.98, 95% CI, 2.32–27.48, *p* = 0.001 and OR 29.75, 95% CI, 4.41–200.94, *p* < 0.0001, respectively, compared to Arabs. In addition, participants who shared a bedroom with 3 or more persons were more likely to have *H. pylori* infection, OR 3.76, 95% CI, 1.41–10, *p* = 0.008 compared to those who shared a bedroom with one or two persons (Table 3).

In term of gastrointestinal characteristics, it was found that participants with *H. pylori* infection were more likely to have abdominal pain (OR 6.87, 95% CI, 2.39–19.79, *p* = 0.001) compared with those of reference level with no gastrointestinal problems. Moreover, it was found that participants with a family history of peptic ulcer were more likely to develop *H. pylori* infection (OR 22.07, 95% CI, 6.01–81.11, *p* < 0.0001) compared to those of reference level with no family history of gastrointestinal problems (Table 3).

## 3. Discussion

The current study has assessed for the first time the prevalence of *H. pylori* in asymptomatic healthy children and adult residents of UAE and the risk factors associated with the infection. Previous studies in the UAE have shown variable prevalence results depending on the purpose of the study. For example, the prevalence of *H. pylori* was found 90% in dyspeptic patients [35,36]. However, in another study evaluating the rate of complications of sleeve gastrectomies, it was found that 44% of symptomatic and asymptomatic patients were *H. pylori*-infected [37]. In another study performed to find an association between diabetes mellitus 2 and *H. pylori* infection, a prevalence of 76.7% was found in diabetic subjects compared to 64.8% in non-diabetic subjects [38]. Only two studies conducted in 2002 and 2006 by Bener et al. have investigated the prevalence of *H. pylori* in asymptomatic UAE patients [33,34]. The first prospective study was conducted on 151 subjects (76 farmers and 75 non-farmers). The prevalence of *H. pylori* was 74.2% when using the IgG *H. pylori* antibody and 51% when using the IgA *H. pylori* antibody [33]. Overall, there was no difference in *H. pylori* prevalence between the UAE asymptomatic farmers and non-farmers. In another study performed on asymptomatic subjects from low socioeconomic background, the *H. pylori* prevalence was found 78.4% in industrial workers and 64.3% in referent workers with a statistical significance between the exposed industrial and non-exposed control groups [34]. In summary, the few studies conducted on *H. pylori* in UAE in the past 26 years presented several limitations and were, therefore, nonrepresentative of the general population in the UAE. The studies were either restricted to dyspeptic subjects or to a special population such as farmers and non-farmers or low socio-economic workers in UAE. None of the performed studies have taken into consideration the asymptomatic and general multinational population residents in UAE including children and adults. Moreover, the serological test used to detect *H. pylori* presented discordant values between the used antibodies IgG and IgA and do not necessarily reveal an active ongoing infection [33]. Therefore, the current prevalence findings are incomparable with the previous studies [33,34].

In our study, the prevalence of *H. pylori* was significantly high. Almost half of the study participants were found to be infected by *H. pylori.* However, the prevalence might be considered low compared to other Arab neighboring countries. In Jordan and Iraq, the prevalence was found to be 77.5% and 78% respectively [39,40]. In Kuwait and Egypt, *H. pylori* was present in 84% and 86% of individuals, respectively [41,42]. In North African countries, the estimated prevalence in Libya, Morocco, and Tunisia were 76%, 75.5%, and 64%, respectively [43,44,45]. In other countries of the Persian Gulf region, the prevalence of *H. pylori* was found 79% in Bahrain [46]. Our estimate of *H. pylori* infection is comparable to the prevalence of the neighboring country Oman (49.1%) but lower than Saudi Arabia (65.9%) [1]. Compared to other countries in the MENA region including Egypt, Libya, Saudi Arabia, Turkey, the prevalence ranged from 50% to 94% in both the children and adult population [1]. In Asia, India and Bangladesh have shown the highest prevalence of *H. pylori*, especially in adults (90% and 88%, respectively). In Africa, Ethiopia has shown the highest prevalence of *H. pylori*. More than 95% of Ethiopian adults were found positive for *H. pylori* [32]. These previous findings may explain why Indian and Ethiopian residents in UAE were more prone to *H. pylori* infection than other nationalities. Interestingly, it was found that nannies presented a higher prevalence of *H. pylori* infection (64%) compared to other professions. Since most of the recruited nannies in this study arrived very recently to UAE, it was hypothesized that most of the infected nannies harbored the *H. pylori* infection from their country of origin. Further studies are needed to explore these findings. Among the recruited nannies, Ethiopian nannies presented the highest prevalence of *H. pylori* (50%) compared to Filipino (44%) and Indonesian nannies (6%). In parallel, the prevalence of *H. pylori* in Emiratis was the highest among the Arab Asian participants. Therefore, prevention of gastric cancer and gastro-duodenal diseases should target these highly prevalent parts of the population of UAE. 

In terms of gender, our findings indicated a statistical difference in the prevalence of *H. pylori* (*p* = 0.001) with women more infected than men (53% vs. 35%). These findings are in disagreement with a recent worldwide *H. pylori* prevalence study which showed no differences in *H. pylori* infection between male and female subjects [47]. The issue of gender disparity in *H. pylori* infection is an intriguing topic and further research is needed to understand the mechanisms by which sex may influence the acquisition and persistence of infection. In the current study, female participants were found more likely to be effected by *H. pylori* infection, possibly because the majority were working as nannies and belonged to low socioeconomic status families. Moreover, they came from highly endemic countries, like Ethiopia. In term of age, our findings showed that the prevalence of *H. pylori* infection increased with age significantly (*p* = 0.032) and the obtained results were in agreement with other performed studies [48,49]. In term of living conditions, participants living in crowded conditions (≥3 members per room) were found to be more prone to the infection by promoting the intrafamilial transmission of *H. pylori.* These findings were in agreement with other study [17,50]. In term of gastrointestinal characteristics, it was found that participants having abdominal pain and family history of peptic ulcer were more prone to *H. pylori* infection. Usually, most peptic ulcer cases are caused by *H. pylori* and since *H. pylori* transmission is intrafamilial, this can explain the high prevalence of *H. pylori* among these participants. 

Apart from the findings of this study, selecting the most suitable test for *H. pylori* detection in this epidemiological study was one of our study challenges. All the diagnostic tests for *H. pylori* tests have different purposes and limitations [20,51]. The choice of Premier Platinum HpSA test in our study depended to a large extent on the noninvasiveness requirement. One of the major advantages of the HpSA test is that it reveals active ongoing *H. pylori* infection and does not require a phlebotomist compared to other serological tests. Serological tests have been used widely in epidemiological studies. However, not all the currently available serological tests have the same accuracy [20,21]. The invasive gold standard method including the endoscopic examination followed by *H. pylori* culture from the gastric biopsy remains the excellent and more reliable test used for *H. pylori* detection. Unfortunately, this method requires medical facilities, equipment on-site, and culture conditions which have rendered the application of this method in epidemiological studies expensive and difficult to perform, especially on children. Moreover, for ethical consideration, it is not possible to use an invasive test for an epidemiological study when noninvasive test exists. Detection of *H. pylori* by molecular techniques such as PCR give the additional possibility to test the antimicrobial susceptibility of *H. pylori* and give important indications for future treatment. However, the PCR method relies in the first place on the availability of the gastric biopsy. Taking together all the limitations of the above-listed tests, it was found that Premier Platinum HpSA was the most appropriate epidemiological tool with high sensitivity and specificity, especially in children [27,28,51]. Several studies using stool *H. pylori* antigen tests have shown that the sensitivity and specificity of stool antigen testing are comparable to histology, culture, or urea breath test [24,27]. However, the main limitations of the HpSA are the invalidity of the test on watery diarrheal stools and on patients who have taken antimicrobial medications, proton pump inhibitors, and bismuth preparations. It was demonstrated that previous exposure to antibiotic therapy for *H. pylori* may result in the conversion of bacillary form to a coccoid form of *H. pylori* which is a morphological manifestation of bacterial cell death without an infective capacity [24], thus HpSA might detect antigens from the degradation of the two morphological forms of *H. pylori* [52]. To overcome such limitations, all watery collected stool samples and volunteer participants under recent antimicrobial medications, bismuth, and potent proton pump inhibitors were excluded from the study. 

The study has provided a comprehensive overview of the prevalence of *H. pylori* in Sharjah, Ajman, Abu Dhabi, and Dubai taking into consideration the multinational UAE residents and the different factors associated with this infection. These data can be used to support national initiatives to prevent and eradicate *H. pylori*, with the goal of reducing the complications of the infection. However, the authors are aware that the current study provided only an overview of the prevalence of *H. pylori* in the UAE. It is considered a pilot study and the obtained results certainly cannot be generalized toward the whole UAE population due to its limitations such as convenience sampling methods, and low sample size and nature of cross-sectional studies which cannot establish causality. More efforts are in process to conduct further large-scale and multicenter epidemiological studies among the seven emirates in UAE.

## 4. Materials and Methods

### 4.1. Ethical Statement

The study was reviewed and approved by the Research and Ethics Committee of the University of Sharjah [REC-17-04-17-01] (see supplementary “Ethical Approval”). All demographic data related to the volunteer participants were recorded and handled with privacy. All volunteer participants signed their consent to participate in the current study. 

### 4.2. Study Design

The cross-sectional study was carried out between September 2017 and April 2018. Four emirates of UAE were included in this prevalence study. A total of 350 stool samples were consecutively collected from healthy asymptomatic children and adult residents in the UAE. Prior to sample collection, the principal investigator made visits to schools, nurseries, housemaid offices, and industrial companies to explain the purpose of the study and to seek consent. To avoid sampling from patients with gastrointestinal disturbances, hospitals and medical centers were excluded. Almost half (48%) of the participants in the study were from Sharjah followed by 33.7% from Abu Dhabi, 15.4% from Ajman and 3% from Dubai. A part-time research assistant was recruited to collect the stool samples and to assist participants in filling out the questionnaire. Resistance to provide a stool sample was one of the challenges encountered. For this reason, the anonymous contribution system was applied during the stool sample collection. To achieve this, two empty boxes were distributed in all collection sites. One box contained the empty stool container and the questionnaire, and the second box was used as a drop box for the stool sample. The part-time research assistant was in charge to check these boxes daily and to aseptically collect and freeze the stool samples at −20 °C. After participants signed the consent form, an enveloped questionnaire was distributed to them along with the stool container. For participating children, parents were requested to sign the consent form and to fill in the questionnaire. The questionnaire and consent forms were written in both English and Arabic. Industrial workers and Ethiopian nannies were assisted by research assistants speaking the same language. For stool sample collection from nurseries and schools, helpers were asked to cooperate with the assigned research assistant. Participants with equivocal questionnaire were taken out of the study. Inclusion criteria were UAE residents aged 1 year and above. Participants subjected to antimicrobials, proton pump inhibitors, and bismuth preparations within the last two weeks from the study period were excluded. The questionnaire consisted of three main sections. The first section consisted of demographic and socioeconomic data which included gender, age, nationality, profession, marital status, education level, family income, number of siblings, number of family members per room, and number of rooms per house. The second section consisted of the lifestyle characteristics of participants. The third section included the gastrointestinal disturbances and family history of *H. pylori* infection. Participants from several nationalities were involved in the study. They were classified into Arab Asians, Non-Arab Asians, and Africans. Arab Asians were considered from the following countries: UAE, Jordan, Iraq, Syria, Lebanon, and Palestine. Non-Arab Asians were considered from India, Pakistan, Afghanistan, the Philippines and Indonesia. Africans were considered from Sudan, Egypt, and Ethiopia. Body mass index (BMI) was classified into four categories: underweight (≤18.5), normal weight (18.5–24.9), overweight (25.0–29.9), and obese (≥30.3).

### 4.3. H. pylori Stool Antigen Test (Premier Platinum HpSA)

The Premier Platinum HpSA is a sandwich enzyme immunoassay which employs a plurality of monoclonal anti-*H. pylori* antibodies absorbed to microwells. The collected stool samples were received in an airtight transport container, then stored either at 4 °C for up to three days before the test or frozen immediately upon received at −20 °C until tested. Diluted stool samples, positive and negative controls were added to each antibody-coated microwell. Tests were performed in duplicate according to the manufacturer’s instructions. Absorbance equal to or above 0.1 was considered a positive result. As the test has not been validated for use on watery diarrheal stools, all diarrheal stool collected were discarded. Volunteers participants subjected to antimicrobials, proton pump inhibitors, and bismuth preparations within the last two weeks were excluded from the study.

### 4.4. Statistical Analysis

Data were analyzed using the IBM SPSS Statistic 25.0 for Windows. The Chi-Square was used to test group differences. Significant predictors of *H. pylori* positivity and socio-demographic, lifestyle characteristics, and gastrointestinal symptoms were assessed using multiple logistic regressions. The significance level was determined at *p*-value < 0.05.

## 5. Conclusions

For the first time, the prevalence of *H. pylori* and its associated risk factors has been evaluated among healthy asymptomatic children and adults residents in the UAE. Almost half of the tested study participants were found to be infected with *H. pylori*, including children and adults. Moreover, univariate and multivariate logistic regression analysis showed a significant association between *H. pylori* infection and gender, age, ethnicity, profession, domestic overcrowding, source of drinking water, and gastrointestinal characteristics of participants. Prevention is worthy by taking into consideration the socio-demographic factors, lifestyle, and gastrointestinal characteristics found to be associated with *H. pylori* infection. Understanding the prevalence pattern of *H. pylori* in UAE will help in prioritizing public health efforts to better manage the complications of *H. pylori* infection.

## Figures and Tables

**Table 1:** Distribution of sample characteristics by place of residence, N = 350.

Characteristic	Place of Residency			
	Abu Dhabi	Dubai	Sharjah	Ajman
**Collection period**	1 September to 20 December 2017	6 January to 6 February 2018	6 January to 30 April 2018	1 March to 30 April 2018
**Locations**	The industrial area of Abu Dhabi- Musaffah, Abu Dhabi City.	Housemaid offices, Private schools, and nurseries.	The University of Sharjah, housemaid offices, private schools, and nurseries.	Ajman soccer club, Housemaid offices, Private schools, and nurseries.
**Participants, n (%)**	118 (33.7)	10 (2.9)	168 (48)	54 (15.4%)
**Gender, n (%)**				
Female	3 (2.3)	5 (3.8)	90 (68.7)	33 (25.2)
Male	115 (52.5)	5 (2.3)	78 (35.6)	21 (9.6)
**Ethnicity, n (%)**				
Arab	3 (1.8)	6 (3.7)	131 (80.4)	23 (14.1)
Asian	115 (69.7)	3 (1.8)	32 (19.4)	15 (9.1)
African	0 (0)	1 (4.5)	5 (22.7)	16 (72.8)
**Age group, n (%)**				
0–1	0	0	13 (100)	0
2–5	0	0	86 (96.6)	3 (3.4)
6–15	1 (2.5)	2 (5)	29 (72.5)	8 (20)
16–30	15 (21.4)	3 (4.3)	23 (32.9)	29 (41.4)
>30	102 (73.9)	5 (3.6)	17 (12.3)	14 (10.1)
**Hp (+), n (%)**	51 (43.2)	1 (10)	58 (34.5)	35 (64.8)

**Table 2:** Bivariate analyses of prevalence, n (%), of *H. pylori* infection according to socio-demographic, lifestyle, and gastrointestinal characteristics, (N = 350).

Variables	Total	HP(−) (n = 205)	Hp(+) (n = 145)	*Chi-Square p-Value*
**Socio-Demographic data**				
**Gender**				
Female	131 (37)	62 (47)	69 (53)	0.001 *
Male	219 (63)	143 (65)	76 (35)	
**Age group**				
0–1	13 (3.7)	9 (69.2)	4 (30.8)	0.032 *
2–5	89 (25.4)	62 (69.7)	27 (30.3)	
6–15	40 (11.4)	26 (65)	14 (35)	
16–30	70 (20)	33 (47.1)	37 (52.9)	
>30	138 (39.4)	75 (54.3)	63 (46)	
**Ethnicity**				
Arab	163 (46.6)	113 (69.3)	50 (30.7)	<0.0001 *
Asian	165 (47.1)	88 (53.3)	77 (46.7)	
African	22 (6.3)	4 (18.2)	18 (81.8)	
**Occupation**				
Professional	37 (10.6)	21 (56.8)	16 (43.2)	0.001 *
Industrial	97 (27.7)	57 (58.8)	40 (41.2)	
Nanny	56 (16)	20 (35.7)	36 (64.3)	
Student	145 (41.4)	94 (64.8)	51 (35.2)	
Unemployed	15 (4.3)	13 (86.7)	2 (13.3)	
**Household income, month/AED**				
≤10,000	133 (45.4)	77 (52.2)	56 (47.8)	0.847
11,000–20,000	25 (7.1)	16 (64)	9 (36)	
>20,000	192 (36)	112 (58.3)	80 (41.7)	
**Number of persons/house**				
1–3	31 (8.9)	16 (51.6)	15 (48.4)	0.676
4–6	207 (59.1)	124 (60)	83 (40)	
≥7	112 (32)	65 (58)	47 (42)	
**Number of persons/room**				
1–2	156 (44.6)	4 (66.7)	2 (33.3)	<0.0001 *
≥3	194 (55.4)	97 (50)	97 (50)	
**Lifestyle characteristics**				
**Contact with domestic animals**				
No	305 (87.1)	181 (59.3)	124 (40.7)	0.445
Yes	45 (12.9)	24 (53.3)	21 (46.7)	
**Source of Drinking water**				
Bottled	283 (80.9)	179 (63.3)	104 (36.7)	0.001 *
Tap	58 (16.6)	24 (41.4)	34 (58.6)	
Boiled	9 (2.6)	7 (77.8)	2 (22.2)	
**Gastrointestinal Characteristics**				
**Gastrointestinal symptoms**				
None	288 (82.3)	179 (62.2)	109 (37.8)	0.001 *
Bloating	20 (5.7)	12 (60)	8 (40)	
Abdominal pain	29 (8.3)	7 (24.1)	22 (75.9)	
Others	13 (3.7)	7 (53.8)	6 (46.2)	
**Parent gastric diseases history**				
None	293 (83.7)	189 (64.5)	104 (35.5)	<0.0001 *
Peptic ulcer	31 (8.9)	3 (9.7)	28 (90.3)	
Others	26 (7.4)	13 (50)	13 (50)	

* Significant at level *p* ≤ 0.05, AED = United Arab Emirates Dirham.

**Table 3:** Multivariate binary logistic regression analysis for factors associated with *H. pylori* infection, (N = 350).

Variable	Odds Ratio (95% Confidence Interval)	*p* Value
**Gender**		
Male	1	
Female	2.52 (1.16–5.50)	0.020 *
**Age groups (years)**		
0–1	1	
2–5	1.67 (0.33–8.39)	0.536
6–15	1.73 (0.31–9.85)	0.534
16–30	1.35 (0.19–9.59)	0.763
>30	1.36 (0.17–10.71)	0.771
**Ethnicity**		
Arab	1	
Asian	7.98 (2.32–27.48)	0.001 *
African	29.75 (4.41–200.94)	<0.0001 *
**Occupation**		
Professional	1	
Industrial	0.42 (0.15–1.15)	0.092
Nanny	2.79 (0.54–14.52)	0.223
Student	0.20 (0.03–1.41)	0.105
Unemployed	0.47 (0.05–4.85)	0.526
**Source of drinking water**		
Bottled	1	
Tap	1.11 (0.31–3.94)	0.875
Boiled	3.17 (0.44–23.08)	0.254
**Number of persons/room**		
1–2	1	
≥3	3.76 (1.41–10.00)	0.008 *
**Parent gastric diseases history**		
None	1	
Peptic ulcer	22.07 (6.01–81.11)	<0.0001 *
Others	1.92 (0.75–4.92)	0.176
**Gastrointestinal symptoms**		
None	1	
Bloating	0.68 (0.20–2.31)	0.536
Abdominal pain	6.87 (2.39–19.79)	<0.001 *
Others	0.90 (0.25–3.28)	0.869

* Significant at *level p ≤* 0.05.

## Supplementary Materials

The following are available online at https://www.mdpi.com/2076-0817/8/2/44/s1.

## References

[1] ReferencesHooi J.K.Y., Lai W.Y., Ng W.K., Suen M.M.Y., Underwood F.E., Tanyingoh D., Malfertheiner P., Graham D.Y., Wong V.W.S., Wu J.C.Y. (2017). Global Prevalence of Helicobacter pylori Infection: Systematic Review and Meta-Analysis. Gastroenterology.

[2] Cover T.L., Blaser M.J. (2009). Helicobacter pylori in health and disease. Gastroenterology.

[3] Muhammad J.S., Sugiyama T., Zaidi S.F. (2013). Gastric pathophysiological ins and outs of helicobacter pylori: A review. J. Pak. Med. Assoc..

[4] International Agency for Research on Cancer (IARC) (1994). Schistosomes, Liver Flukes and Helicobacter Pylori.

[5] Carrasco G., Corvalan A.H. (2013). Helicobacter pylori-Induced Chronic Gastritis and Assessing Risks for Gastric Cancer. Gastroenterol. Res. Pract..

[6] Uemura N., Okamoto S., Yamamoto S., Matsumura N., Yamaguchi S., Yamakido M., Taniyama K., Sasaki N., Schlemper R.J. (2001). Helicobacter pylori infection and the development of gastric cancer. New Engl. J. Med..

[7] Graham D.Y. (2015). Helicobacter pylori update: gastric cancer, reliable therapy, and possible benefits. Gastroenterology.

[8] Muhammad J.S., Eladl M.A., Khoder G. (2019). Helicobacter pylori-induced DNA Methylation as an Epigenetic Modulator of Gastric Cancer: Recent Outcomes and Future Direction. Pathogens.

[9] Yeh J.M., Kuntz K.M., Ezzati M., Goldie S.J. (2009). Exploring the cost-effectiveness of Helicobacter pylori screening to prevent gastric cancer in China in anticipation of clinical trial results. Int. J. Cancer.

[10] Takenaka R., Okada H., Kato J., Makidono C., Hori S., Kawahara Y., Miyoshi M., Yumoto E., Imagawa A., Toyokawa T. (2007). Helicobacter pylori eradication reduced the incidence of gastric cancer, especially of the intestinal type. Aliment. Pharmacol. Ther..

[11] Bray F., Ferlay J., Soerjomataram I., Siegel R.L., Torre L.A., Jemal A. (2018). Global cancer statistics 2018: GLOBOCAN estimates of incidence and mortality worldwide for 36 cancers in 185 countries. CA Cancer J. Clin..

[12] Ferlay J., Shin H.R., Bray F., Forman D., Mathers C., Parkin D.M. (2010). Estimates of worldwide burden of cancer in 2008: GLOBOCAN 2008. Cancer J. Clin..

[13] Ferro A., Peleteiro B., Malvezzi M., Bosetti C., Bertuccio P., Levi F., Negri E., La Vecchia C., Lunet N. (2014). Worldwide trends in gastric cancer mortality (1980–2011), with predictions to 2015, and incidence by subtype. Eur. J. Cancer.

[14] Schulz T.R., McBryde E.S., Leder K., Biggs B.A. (2014). Using stool antigen to screen for Helicobacter pylori in immigrants and refugees from high prevalence countries is relatively cost effective in reducing the burden of gastric cancer and peptic ulceration. PloS ONE.

[15] Muhammad J.S., Zaidi S.F., Saeed S.A., Ishaq M. (2017). Current status of Helicobacter pylori association with haematological and cardiovascular diseases: A mini review. J. Pak. Med. Assoc..

[16] Schwarz S., Morelli G., Kusecek B., Manica A., Balloux F., Owen R.J., Graham D.Y., van der Merwe S., Achtman M., Suerbaum S. (2008). Horizontal versus familial transmission of Helicobacter pylori. PLoS Pathog..

[17] Forman D., De Backer G., Elder J., Moller H., Damotta L.C., Roy P., Abid L., Tjonneland A., Boeing H., Haubrich H. (1993). Epidemiology of, and risk factors for, Helicobacter pylori infection among 3194 asymptomatic subjects in 17 populations. Gut.

[18] Graham D.Y., Malaty H.M., Evans D.G., Evans D.J., Klein P.D., Adam E. (1991). Epidemiology of Helicobacter pylori in an asymptomatic population in the United States. Effect of age, race, and socioeconomic status. Gastroenterology.

[19] Venneman K., Huybrechts I., Gunter M.J., Vandendaele L., Herrero R., Van Herck K. (2018). The epidemiology of Helicobacter pylori infection in Europe and the impact of lifestyle on its natural evolution toward stomach cancer after infection: A systematic review. Helicobacter.

[20] Cutler A.F., Havstad S., Ma C.K., Blaser M.J., Perez-Perez G.I., Schubert T.T. (1995). Accuracy of invasive and noninvasive tests to diagnose Helicobacter pylori infection. Gastroenterology.

[21] Burucoa C., Delchier J.C., Courillon-Mallet A., de Korwin J.D., Megraud F., Zerbib F., Raymond J., Fauchere J.L. (2013). Comparative evaluation of 29 commercial Helicobacter pylori serological kits. Helicobacter.

[22] Vaira D., Malfertheiner P., Megraud F., Axon A.T., Deltenre M., Gasbarrini G., O’Morain C., Pajares Garcia J.M., Quina M., Tytgat G.N., The European Helicobacter pylori HpSA Study Group (2000). Noninvasive antigen-based assay for assessing Helicobacter pylori eradication: A European multicenter study. Am. J. Gastroenterol..

[23] Megraud F., Bessede E., Lehours P. (2014). Diagnosis of Helicobacter pylori infection. La Rev. Du Prat..

[24] Vaira D., Malfertheiner P., Megraud F., Axon A.T. (1999). Diagnosis of Helicobacter pylori infection by HpSA test. Lancet.

[25] van Doorn O.J., Bosman D.K., van’t Hoff B.W., Taminiau J.A., ten Kate F.J., van der Ende A. (2001). Helicobacter pylori Stool Antigen test: a reliable non-invasive test for the diagnosis of Helicobacter pylori infection in children. Eur. J. Gastroenterol. Hepatol..

[26] Moubri M., Burucoa C., Kalach N., Larras R.R., Nouar N., Mouffok F., Arrada Z. (2018). Performances of the IDEIA HpStAR Stool Antigen Test in Detection of Helicobacter pylori Infection Before and After Eradication Treatment in Algerian Children. J. Trop. Pediatrics.

[27] Queiroz D.M., Saito M., Rocha G.A., Rocha A.M., Melo F.F., Checkley W., Braga L.L., Silva I.S., Gilman R.H., Crabtree J.E. (2013). Helicobacter pylori infection in infants and toddlers in South America: concordance between [13C] urea breath test and monoclonal H. pylori stool antigen test. J. Clin. Microbiol..

[28] Nguyen T.V., Bengtsson C., Nguyen G.K., Granstrom M. (2008). Evaluation of a novel monoclonal-based antigen-in-stool enzyme immunoassay (Premier Platinum HpSA PLUS) for diagnosis of Helicobacter pylori infection in Vietnamese children. Helicobacter.

[29] Koletzko S., Konstantopoulos N., Bosman D., Feydt-Schmidt A., van der Ende A., Kalach N., Raymond J., Russmann H. (2003). Evaluation of a novel monoclonal enzyme immunoassay for detection of Helicobacter pylori antigen in stool from children. Gut.

[30] World Gastroenterology O. (2011). World Gastroenterology Organisation Global Guideline: Helicobacter pylori in developing countries. J. Clin. Gastroenterol..

[31] Burucoa C., Axon A. (2017). Epidemiology of Helicobacter pylori infection. Helicobacter.

[32] Hunt R.H., Xiao S.D., Megraud F., Leon-Barua R., Bazzoli F., van der Merwe S., Vaz Coelho L.G., Fock M., Fedail S., Cohen H. (2011). Helicobacter pylori in developing countries. World Gastroenterology Organisation Global Guideline. J. Gastrointest. Liver Dis..

[33] Bener A., Uduman S.A., Ameen A., Alwash R., Pasha M.A., Usmani M.A., SR A.I.-N., Amiri K.M. (2002). Prevalence of Helicobacter pylori infection among low socio-economic workers. J. Commun. Dis..

[34] Bener A., Adeyemi E.O., Almehdi A.M., Ameen A., Beshwari M., Benedict S., Derballa M.F. (2006). Helicobacter pylori profile in asymptomatic farmers and non-farmers. Int. J. Environ. Health Res..

[35] Adeyemi E.O., Fadlalla H., al-Homsi M., Nnalue N.A., Goodwin S., Boehme D., Sim A.J. (1992). Clinicopathological assessment of gastric biopsy samples of patients with Helicobacter pylori infection--metronidazole resistance and compliance problems in the United Arab Emirates. Ital. J. Gastroenterol..

[36] Zaitoun A.M. (1994). Histological study of chronic gastritis from the United Arab Emirates using the Sydney system of classification. J. Clin. Pathol..

[37] Albawardi A., Almarzooqi S., Torab F.C. (2013). Helicobacter pylori in sleeve gastrectomies: prevalence and rate of complications. Int. J. Clin. Exp. Med..

[38] Bener A., Micallef R., Afifi M., Derbala M., Al-Mulla H.M., Usmani M.A. (2007). Association between type 2 diabetes mellitus and Helicobacter pylori infection. Turk. J. Gastroenterol. Off. J. Turk. Soc. Gastroenterol..

[39] Nimri L.F., Matalka I., Bani Hani K., Ibrahim M. (2006). Helicobacter pylori genotypes identified in gastric biopsy specimens from Jordanian patients. BMC Gastroenterol..

[40] Hussein N.R., Robinson K., Atherton J.C. (2008). A study of age-specific Helicobacter pylori seropositivity rates in Iraq. Helicobacter.

[41] Al Qabandi A., Mustafa A.S., Siddique I., Khajah A.K., Madda J.P., Junaid T.A. (2005). Distribution of vacA and cagA genotypes of Helicobacter pylori in Kuwait. Acta Trop..

[42] Korstanje A., den Hartog G., Biemond I., Lamers C.B. (2002). The serological gastric biopsy: A non-endoscopical diagnostic approach in management of the dyspeptic patient: Significance for primary care based on a survey of the literature. Scand. J. Gastroenterol. Suppl..

[43] Bakka A.S., Salih B.A. (2002). Prevalence of Helicobacter pylori infection in asymptomatic subjects in Libya. Diagn. Microbiol. Infect. Dis..

[44] Mansour K.B., Keita A., Zribi M., Masmoudi A., Zarrouk S., Labbene M., Kallel L., Karoui S., Fekih M., Matri S. (2010). Seroprevalence of Helicobacter pylori among Tunisian blood donors (outpatients), symptomatic patients and control subjects. Gastroenterol. Clin. Et Biol..

[45] Benajah D.A., Lahbabi M., Alaoui S., El Rhazi K., El Abkari M., Nejjari C., Amarti A., Bennani B., Mahmoud M., Ibrahimi S.A. (2013). Prevalence of Helicobacter pylori and its recurrence after successful eradication in a developing nation (Morocco). Clin. Res. Hepatol. Gastroenterol..

[46] Fakhro A.R., Fateha Bel D., Amin Farid I.M., Jamsheer H.M. (1999). The association between Helicobacter pylori infection and lymphoid reaction in patients suffering from dyspepsia in Bahrain. Saudi J. Gastroenterol. Off. J. Saudi Gastroenterol. Assoc..

[47] Zamani M., Ebrahimtabar F., Zamani V., Miller W.H., Alizadeh-Navaei R., Shokri-Shirvani J., Derakhshan M.H. (2018). Systematic review with meta-analysis: the worldwide prevalence of Helicobacter pylori infection. Aliment. Pharmacol. Ther..

[48] Wangda S., Richter J.M., Kuenzang P., Wangchuk K., Choden T., Tenzin K., Malaty H.M. (2017). Epidemiology of Helicobacter pylori infection in asymptomatic schoolchildren in Bhutan. Helicobacter.

[49] Toscano E.P., Madeira F.F., Dutra-Rulli M.P., Gonçalves L.O.M., Proença M.A., Borghi V.S., Cadamuro A.C.T., Mazzale G.W., Acayaba R., Silva A.E. (2018). Epidemiological and Clinical-Pathological Aspects of Helicobacter pylori Infection in Brazilian Children and Adults. Gastroenterol. Res. Pract..

[50] Galpin O.P., Whitaker C.J., Dubiel A.J. (1992). Helicobacter pylori infection and overcrowding in childhood. Lancet.

[51] Kabir S. (2001). Detection of Helicobacter pylori in faeces by culture, PCR and enzyme immunoassay. J. Med. Microbiol..

[52] Kusters J.G., Gerrits M.M., Van Strijp J.A., Vandenbroucke-Grauls C.M. (1997). Coccoid forms of Helicobacter pylori are the morphologic manifestation of cell death. Infect. Immun..

