# Supplementary material for: Prevalence of Helicobacter pylori and Its Associated Factors among Healthy Asymptomatic Residents in the United Arab Emirates
Source: Pathogens. 2019 Apr 1;8(2):44. doi: 10.3390/pathogens8020044 (PMC6632043; doi:10.3390/pathogens8020044)
Supplement: Supplementary file 1 [file pathogens-08-00044-s001.zip › Questionnaire.pdf]

Questionnaire number:

_____

جامعة الشارقة
UNIVERSITY OF SHARJAH

Prevalence of *H. pylori* and its associated risk factors

The aim of this questionnaire is to study the prevalence of *H. pylori* and its associated risk factors. The study will help enormously in the prevention and treatment of the severe gastric diseases caused by *H. pylori* infection. The study will be conducted by Dr. Ghalia Khoder, assistant professor at College of Pharmacy, University of Sharjah. To perform the study, we require from you as volunteer a stool sample and the below filled questionnaire.

Do you accept to participate as a volunteer in this study ? Yes ☐ No ☐

1. Sex : Female ☐ Male ☐

2. Age : ________years or _________months (for baby)

3. Nationality : __________________

4. Weight : _________kg and height _________m

5. Profession : ___________

6. Place of residency: Abu Dhabi ☐ Dubai ☐ Sharjah ☐ RAK ☐ Ajman ☐ Fujairah ☐ Umm al Quwain ☐

7. Marital status : Not married ☐ Married ☐ Widow ☐ Divorced ☐

8. Education level : No formal education ☐ Primary ☐ Secondary ☐ University ☐

9. Smoking history:

	Yes	No
Cigarette	☐	☐
Shisha	☐	☐

In case you are smoking shisha, do you share with others the same shisha`s accessories ? Yes ☐ No ☐

10. Total family income:

- ☐ Less than 5 000 AED /family
- ☐ 5 000 – 10 0000 AED / family
- ☐ 10 000- 20 000 AED / family
- ☐ 20 000 -30 000 AED /family
- ☐ More than 30 000 AED /family
- ☐ No family income

11. Number of family members per house:

1-3	4-6	7-9	More than 10
☐	☐	☐	☐

12. Number of rooms per house :

1 room	2 rooms	3 rooms	More than 3 rooms
☐	☐	☐	☐

13. Number of family members per room :

1	2	3	4	More than 4
☐	☐	☐	☐	☐

14. Do you have contact with any domestic animals ? Yes ☐ No ☐

If your answer is yes, which type of domestic animals do you usually contact ?

Cat	Dog	Cow	Camel	Sheep	Bird	Other
☐	☐	☐	☐	☐	☐	☐

15. Do you suffer from any of the below listed diseases?

	Yes	No	Not sure
Diabetes	☐	☐	☐
Hypertension	☐	☐	☐
Anemia	☐	☐	☐
Asthma	☐	☐	☐
Kidney failure	☐	☐	☐
cancer	☐	☐	☐

16. What is your frequent source of drinking water ?

Tap water	Mineral bottled water	Boiled water	Well
☐	☐	☐	☐

17. How often are you eating the below nutrional products ?

	Never / Rarely	Sometimes/ often
Poultry products	☐	☐
Raw vegetables	☐	☐
Fish products	☐	☐
Raw meat	☐	☐

18. Do you suffer from any gastrointestinal pain ? Yes ☐ No ☐

If your answer is yes, from which of the below gastrointestinal pain are you suffering frequently ?

Blotting	Nausea	Vomiting	Abdominal pain	Burning sensation	others
☐	☐	☐	☐	☐	☐

19. Did any of your parents suffer from severe gastric diseases ? Yes ☐ No ☐

If your answer is yes, from which gastric diseases have they suffered ?

Peptic ulcer	Gastric cancer	MALT lymphoma	Other gastric diseases
☐	☐	☐	☐

20. How often are you using the following drugs ?

	Never / Rarely	Sometimes/ often
Vitamin	☐	☐
Aspirin	☐	☐
Panadol/Paracetmol	☐	☐
Proton Pump inhibitor	☐	☐

21. Are you currently under *H. pylori* eradication treatment such as proton pump inhibitors, antibiotics or bismuth ? Yes ☐ No ☐

22. Did you take any *H. pylori* eradication treatment such as proton pump inhibitors, antibiotics or bismuth for the last two weeks ? Yes ☐ No ☐
