# Supplementary material for: Prevalence of Helicobacter pylori and Its Associated Factors among Healthy Asymptomatic Residents in the United Arab Emirates
Source: Pathogens. 2019 Apr 1;8(2):44. doi: 10.3390/pathogens8020044 (PMC6632043; doi:10.3390/pathogens8020044)
Supplement: Supplementary file 1 [file Ethical Approval.pdf › Ethical Approval.pdf]

Office of Vice Chancellor for
Research & Graduate Studies
Research Ethics Committee

جامعـــــة الشـــــارقة
UNIVERSITY OF SHARJAH

مكتب نائب مدير الجامعة لشؤون
البحث العلمي والدراسات العليا
لجنة أخلاقيات البحث العلمي

- **Date:** 04 / 06 / 2017
- **Reference number:** REC-17-04-17-01
- **Title of the research:**

  Helicobacter pylori Infection : Prevalence and Risk Factors

- **Name of the principle investigator:** Dr. Ghalia khoder

Dear Dr. Ghalia,

The Research Ethics Committee has reviewed the above application, and has voted in favor of approving it from an ethical perspective.

Kindly note that this approval is based on the conditions that,

1. the research is executed according to the research protocol described in the application form, and/or its subsequent modifications, if those modifications were requested by the Research Ethics Committee
2. the information sheet and/or informed consent are those approved by the Research Ethics Committee
3. the research tools are those approved by the Research Ethics Committee

Please note that it is your responsibility, as the principle investigator, to immediately inform the Committee of any changes in the research protocol and/or the research methodologies, should the need for those changes arise prior to or during the conduct of this research study.

On behalf of the Research Ethics Committee, I wish you and your team success in your research project.

Sincerely,

Dr. Suhail Al-Amad
Chair, Research Ethics Committee
University of Sharjah

Tel: +971-6-505-3004 Fax: +971-6-505-3011 P.O.BOX: 27272, Sharjah, United Arab Emirates
هاتف:97165053001+ فاكس: 97165053011+ ص.ب: 27272- الشارقة-الإمارات العربية المتحدة
VCRGS@sharjah.ac.ae www.Sharjah.ac.ae
